# Inhibition of lung cancer growth and metastasis by DHA and its metabolite, RvD1, through miR-138-5p/FOXC1 pathway

**DOI:** 10.1186/s13046-019-1478-3

**Published:** 2019-11-29

**Authors:** Xiaoming Bai, Jiaofang Shao, Sujin Zhou, Zhenggang Zhao, Fanghong Li, Rong Xiang, Allan Z. Zhao, Jinshun Pan

**Affiliations:** 1 Department of Pathology, Nanjing Medical University, Nanjing, 210029 People’s Republic of China; 2 Department of Bioinformatics, Nanjing Medical University, Nanjing, 210029 People’s Republic of China; 3 School of Biomedical and Pharmaceutical Sciences, Guangdong University of Technology, Guangzhou, 510006 People’s Republic of China; 4 Department of Pathology, The Second People’s Hospital of Nantong, Nantong, 226000 People’s Republic of China; 5 Department of Biotherapy, The Second Affiliated Hospital, Nanjing Medical University, Nanjing, 210011 People’s Republic of China

**Keywords:** Resolvin D1, miR-138-5p, FOXC1, Growth and invasion, Lung cancer

## Abstract

**Background:**

Non small cell lung cancer (NSCLC) is one of the most common cancers in the world. DHA is known to be capable of suppressing NSCLC cell proliferation and metastasis. However, the mechanisms by which DHA exhibits its antitumor effects are unknown. Here we aimed to identify the effects and mechanisms of DHA and its metabolites on lung cancer cell growth and invasion.

**Methods:**

As measures of cell proliferation and invasion ability, the cell viability and transwell assays were used in vitro. Transgenic mfat-1 mice, which convert ω-6 PUFAs to ω-3 PUFAs, were used to detect the effect of endogenous DHA on tumor transplantation. An LC − MS/MS analysis identified the elevation of several eicosanoid metabolites of DHA. By using qPCR miRNA microarray, online prediction software, luciferase reporter assays and Western blot analysis, we further elucidated the mechanisms.

**Results:**

Addition of exogenous DHA inhibited the growth and invasion in NSCLC cells in vitro. Endogenously produced DHA attenuated LLC-derived tumor growth and metastasis in the transgenic mfat-1 mice. Among the elevation of DHA metabolites, resolvin D1 (RvD1) significantly contributed to the inhibition in cell growth and invasion. MiRNA microarray revealed that the level of miR-138-5p was significantly increased after RvD1 treatment. MiR-138-5p mimics decreased cell viability and invasion; while miR-138-5p inhibitor abolished RvD1-mediated suppression of cell viability and invasion. The expression of FOXC1 was significantly reduced upon overexpression of miR-138-5p while luciferase reporter assay showed that FOXC1 was a direct target of miR-138-5p. In vivo, endogenous DHA by the mfat-1 transgene enhanced miR-138-5p expression and decreased FOXC1 expression. Furthermore, overexpression of FOXC1 reversed the inhibition in cell viability and invasion induced by RvD1 treatment.

**Conclusions:**

These data identified the RvD1/miR-138-5p/FOXC1 pathway as a novel mechanism by DHA and its metabolite, RvD1, and the potential of targeting such pathway as a therapeutic strategy in treating NSCLC.

## Background

Lung cancer is the most common cancer in both incidence and death rate, with non-small cell lung cancer (NSCLC) and small-cell lung cancer accounting for 85 and 15% of the reported cases, respectively [1–3]. Despite advances in detection and improvements to standard of care, NSCLC is often diagnosed at an advanced stage and bears poor prognosis [1]. Although a combination of resection and chemotherapy as well as immunotherapy can improve survival, the prognosis of lung cancer is still poor [4].

Docosahexaenoic acid (DHA), an ω-3 polyunsaturated fatty acid (PUFA), is an essential fatty acid [5]. Epidemiological literatures, including cross-sectional and migrational studies, have revealed a protective effect of DHA against the development of various cancers [6–8]. In NSCLC, DHA not only inhibits cancer cell proliferation and migration [9], but also suppresses angiogenesis [10]. Several DHA metabolites have been found to participate in the control of carcinogenesis [10]. For example, resolvin D1 (RvD1), one of DHA metabolites, has been reported to suppress tumor growth in several murine cancer cell lines at the doses 10,000 times lower than DHA [11]. The potential effects and underlying mechanisms of RvD1 on NSCLC cell growth and invasion, however, are completely unknown, apart from inhibiting TGF-β1-induced epithelial mesenchymal transition in lung cancer [12, 13].

MiRNAs are small, endogenous noncoding RNAs (18–23 nucleotides) that post-transcriptionally regulate gene expression by targeting the 3′-untranslated regions (3′-UTR) of mRNAs [14]. Aberrant expression of some miRNAs has been reported to be associated with malignant phenotypes in various cancers, participating in not only cell proliferation, but also invasion and metastasis [15, 16]. In addition, several miRNAs were involved in the control of cancer cell growth and invasion by DHA metabolites [9, 13]. Among them, miR-138-5p is postulated as a tumor suppressor. Accordingly, the expression of miR-138-5p is decreased in many cancer tissues, compared with adjacent noncancerous tissues; and reduced expression of miR-138-5p is significantly correlated with patients’ clinicopathological factors and poor survival [17, 18]. Among NSCLC cases, miR-138-5p is involved in the elevation of GADD45A and inhibits cell growth [19]. However, little is known about the relationship between DHA and miR-138-5p.

A series of recent studies involving the usage of a transgenic genetic model, fat-1 (or mfat-1) transgenic mice, have shed new light into the mechanisms underlying the protective effects of ω-3 PUFAs [20]. The fat-1 transgenic mice, initially generated in Kang’s lab [21, 22] (and later the mfat-1 mouse model independently designed by our group [23]) carry a globally expressed *C. elegans* fat-1 transgene that encodes an ω-3 fatty acid desaturase. The FAT-1 enzyme can convert ω-6 PUFAs to the corresponding ω-3 forms by adding a double bond to the ω-3 position, thus allowing endogenous production of ω-3 PUFAs while reducing the ω-6: ω-3 ratio in tissues without special dietary adjustment [23, 24]. The fat-1 transgenic model allows researchers to bypass the traditional lengthy dietary approach of feeding fish-oil to the animals, and have been widely applied to the studies related to autoimmune diseases, tumorigenesis, metabolic and cardiovascular diseases, as well as neurological diseases.

In current studies, we found that endogenous DHA produced by mfat-1 gene significantly inhibited cell viability and metastasis, and increased RvD1 production in lung cancer cells. Further, miR-138-5p was upregulated in RvD1-treated lung cancer cells. Herein, we intend to advance the role and mechanisms of DHA and RvD1 on NSCLC growth and metastasis, with the primary focus on miR-138-5p-mediated pathway.

## Materials and methods

### Materials

Dulbecco’s modified Eagle’s medium (DMEM) and Lipofectamine 2000 were obtained from Invitrogen (Carlsbad, CA, USA). DHA, EPA, PUFA analytical standards and Matrigel were obtained from Sigma-Aldrich (St. Louis, MO, USA). The protein assay was from Bio-Rad (Hercules, CA, USA). Water-soluble tetrazolium (WST) reagent was from Dojindo Laboratories (Kumamoto, Japan). Electrochemiluminescence (ECL) reagents were obtained from Amersham Biosciences (Piscataway, NJ, USA). The dual-luciferase reporter assay system was obtained from Promega Corporation (Madison, WI, USA). PrimeScript RT Reagent Kit was obtained from TAKARA Bio Inc. (#RR037A, Shiga, Japan). SYBR Green Master was from Roche Diagnostics (#04913914001, Indianapolis, IN, USA). Resolvin D1 (RvD1) was obtained from Cayman Chemical Co. (#10012554, Ann Arbor, Michigan, USA). The following were commercially obtained antibodies: the anti-Akt (#9272), anti-phospho-Akt (Ser473, #4060), the anti-Erk1/2 (#4696), and anti-phospho-Erk1/2 (Thr202/Tyr204, #8544) antibodies were obtained from Cell Signaling Technology (Danvers, MA, USA); the anti-FOXC1 antibody (#115201) was obtained from Abcam plc (Cambridge, UK); the anti-GAPDH antibody was obtained from Bioworld Technology (Atlanta, Georgia30,305, USA). EnVision+single reagents (Mouse, Rabbit) were from DAKO (K4000, K4002, Glostrup, Denmark).

### Cell culture and reagents

Human lung cancer cell lines (A549 and H1299) and mouse lung cancer cell line (LLC) were cultured in Dulbecco’s modified Eagle’s medium (DMEM; Gibco) supplemented with 10% fetal bovine serum (FBS; Gibco), 100 U/ml penicillin, 100 ng/ml streptomycin. Human embryonic kidney 293 T (HEK293 T) cells were maintained in DMEM supplemented with 10% FBS, 100 U/ml penicillin, 100 ng/ml streptomycin. All cell lines were maintained in a 37 °C incubator with 5% CO2.

### Animals

All animals (female C57BL/6 J and mfat-1 mice) were treated in accordance with the guidelines of the Institutional Animal Care and Use Committee at Nanjing Medical University. The animals were fed regular diet and water ad libitum, and housed at 22 °C with a 12-h light-dark cycle. The normal diet was from Xietong Biotechnology Co. Ltd. (Jiangsu, China). All animal studies comply with the ARRIVE guidelines. The mfat-1 transgenic mice were described in our previous studies [7]. Analyses of ω-6 and ω-3 PUFA composition in transgenic mice were shown in Additional file 1: Table S1. All above strains of mice have a C57BL/6 genetic background.

### Patients and specimens

Primary surgical specimens were collected from 60 patients (aged from 52 to 83; average, 64) who were diagnosed clinically for squamous cell carcinoma (LUSC) or adenocarcinoma (LUAC), from the Jiangsu Province Hospital and the Nanjing Chest Hospital. None of them had distant metastasis. All of them were approached for participation in the project and the experimental protocols were approved by the Human Ethics Committee of Nanjing Medical University, including any relevant details. Written informed consent was obtained from all the donors for use of these samples in research. The work conforms to the provisions of the Declaration of Helsinki in 1975. Resected specimens were fixed with neutral buffered 10% formalin and embedded in paraffin blocks. The diagnosis and histological grade of all the cases were confirmed independently by two pathologists based on World Health Organization (WHO) classification.

### Plasmid transfections

The pCMV-based plasmid encoding human FOXC1 was obtained from the nonprofit plasmid repository (Addgene, MA 02139 USA). A549 cells (2 × 10^5^) were seeded and grown in 6-well culture plates for 24 h before transfection with the plasmids, or empty vector control (2 μg) using Lipofectamine 2000 (5 μl). The efficiency of transfection was assayed by Western blot. The levels of FOXC1 overexpression were evaluated in Additional file 2: Figure S1.

### MiRNAs mimics and siRNA transfection

The miR-138-5p mimics, miR-138-5p inhibitor, and the siRNA targeting FOXC1 (siFOXC1) were obtained from Santa Cruz Biotechnology (Santa Cruz, CA, USA). A549 cells (2 × 10^5^) were plated in 6-well plates and cultured for 24 h. The cells were then transfected with different RNAs using Lipofectamine 2000. After 48 h, the levels of target proteins were confirmed by Western blot, and the cells were subsequently used for further experiments. The levels of overexpression or inhibition of miR-138 were evaluated by qRT-PCR. The levels of inhibition of FOXC1 expression were determined by Western blot. **P < 0.01 compared to corresponding control groups (Additional file 2: Figure S1).

### Overexpression of mfat-1 by lentiviral transfection

The mfat-1 cDNA, as described previously [7], was synthesized and cloned into the PLJM1 lentivirus vector (Addgene, Palo Alto, CA, USA). PLJM1-mfat-1 or the vehicle plasmids were transfected into HEK293T cells, according to manufacturer’s instructions. Lentivirus-containing supernatant was harvested 48 h after transfection and used to infect LLC cells.

### Total RNA isolation, reverse transcription PCR (RT-PCR) and quantitative real-time PCR analysis

Total RNA was isolated from cells using the Trizol reagent, according to the manufacturer’s instructions. For each sample, 0.5 μg of RNA was reverse transcribed using the PrimeScript RT Reagent Kit. For the purposes of the RT-PCR or qPCR analysis, GAPDH was used as the reference.

RT-PCR analysis was used to detect the mfat-1 expression in LLC cells and transgenic mice. Forward primer (5′ -GGACCTGGTGAAGAGCATCCG- 3′) and reverse primer (5′ -GCCGTCGCAGAAGCCAAAC- 3′) were used to detect mfat-1 mRNA, and an amplified fragment of 438 bp was indicative of the mfat-1 gene (Additional file 2: Figure S1).

Real time PCR analysis was performed using the Power SYBR Green PCR master mix. PCR reaction conditions were: pre-incubation at 95 °C for 10 min (1 cycle); 95 °C for 15 s, 60 °C for 15 s and 72 °C for 30 s (40 cycles). Fluorescence emission data were collected during the annealing step. All treatments and conditions were performed in triplicate to calculate statistical significance.

### Western blot

A549 and LLC cells were treated with pharmacological agents for different length as indicated in the figure legends. The cells were collected into lysis buffer and then cleared by centrifugation at 12,000×g for 15 min at 4 °C. Equal amounts of total proteins (20 μg) were separated by SDS-PAGE, detected by a standard enhanced-chemiluminescent reaction and analyzed using Image Lab 4.0 analysis software from Bio-Rad.

### Cell proliferation and invasion assays

Cell proliferation assays were performed in 96-well plate. The cells were stained with WST at 37 °C for 1 h and quantified by the absorbance at 450 nm. Cell invasion assays were performed in 12-well transwell units. After incubation at 37 °C for 24 h, the cells were fixed with ethanol and then stained with 0.1% crystal violet. The cells that had invaded to the lower surface of the membrane were photographed by a LeiCa Microscope. Low high power views (200×) were selected randomly from each sample in a blind manner. The cells invaded to lower surface of transwell units were solubilized with 10% acetic acid and quantified by measuring the absorbance at 590 nm.

### Gas chromatography analysis of fatty acid compositions

Lipids extraction from cells and tissues were performed according to a previous report [7]. The gas chromatography was done on an Agilent 7890A. Identification of components was done by comparison of retention times with those of PUFA analytical standard.

### Metabolomic analysis of eicosanoids

The LLC cells were seeded in 15 cm dish 24 h before adding DHA. 2 × 10^7^ cells were needed for eicosanoid analysis in each sample. Eicosanoid extraction was performed according to a previous report [7]. Chromatographic separation involved an ACQUITY UPLCBEH C18 column consisting of ethylene-bridged hybrid particles. The metabolites were quantified by use of a 5500 QTRAP hybrid triple quadrupole linear ion trap mass spectrometer equipped with a Turbo Ion Spray electrospray ionization (ESI) source.

### Tumor allografts models

Four-week-old female WT and mfat-1 mice were injected with 2 × 10^6^/0.1 ml of LLC cell lines into the flanks subcutaneously. Bi-dimensional tumor measurements were taken every 2 days. Tumor volume was measured along two major axes using callipers. Tumor volume (mm^3^) was calculated as follows: V = 1/2 L × W^2^ (L: length, W: width). Twenty-four days after cancer cells subcutaneous injection, the mice were executed, and the tumor allografts were removed. The tail vein injection was carried out in WT and mfat-1 mice with 5 × 10^5^/0.3 ml of LLC cells. Forty days after tail vein injection, the mice were executed. The lungs and other organs were removed. The specimens were fixed with neutral buffered 10% formalin and embedded in paraffin blocks. Sections (4 μm) of the tumor blocks were used for in situ hybridization and immunohistochemical staining.

### In situ hybridization assays

The RNAscope probe for human and mouse miR-138-5p was hybridized on 4 μm slides of human lung cancer masses and mouse tumor allografts following the in situ hybridization assay protocol. Sequential slides were stained with a mouse-specific control probe following the standard protocol. Four high power views (400×) were selected randomly from each sample in a blind manner; the level of integrated optical density was estimated using the Image Pro Plus software and presented as mean ± SEM.

### Immunohistochemical staining

The sections (4 μm) of tumor blocks were used for immunohistochemical analysis. Sections were treated with primary anti-FOXC1 antibody with PBS used as a negative control. The slices were photographed by under a LeiCa Microscope and Image Analyze system. Four high power views (400×) were selected randomly from each sample in a blind manner; the level of integrated optical density was estimated using the Image Pro Plus software and presented as mean ± SEM.

### Dual-luciferase reporter assay

The 3′-UTR-luciferase reporter constructs containing the 3′-UTR region of FOXC1 with the wild-type and mutant binding sites of miR-138-5p were amplified using PCR. The mutant 3′-UTR construct was made by introducing the mismatch mutation into the putative seed regions of FOXC1. A549 cells (1.0 × 10^5^/well) were seeded in cultured for 24-h in 24-well plates. The cells were then co-transfected with either wild-type (WT1 and WT2) or mutant-type (mut1 and mut2) luciferase reporter plasmids, and equal amounts of miR-138-5p using Lipofectamine2000 according the manufacturer’s instruction. Luciferase activities were measured 24 h after transfection using the Dual Luciferase Reporter Assay System (Promega). Experiments were performed with three independent replicates.

### Statistical analysis

Statistical analysis of the integrated optical density level of pictures was performed using STATA se12.0 software (Stata Corp, Collage Station, TX, USA). The miR-138-5p miRNA expression data and FOXC1 expression data were downloaded from the TCGA database of LUSC and LUAC cancer tissues. The overall survival (OS) was analyzed by the COX Regression Test. The correlation between gene expression and tumor extent (T1-T4), lymph node metastasis (N0-N3) or distant metastasis (M0-M1) was analyzed by Kruskal-Wallis Test or Wilcoxon Test. *P* value was shown. Other data are presented as mean ± SEM. *P*-values were calculated by one-way ANOVA for unpaired samples using the GraphPad Prism software. The results were considered significant at *P < 0.05, **P < 0.01.

## Results

### DHA decreases NSCLC cell growth and invasion

**Fig. 1:**
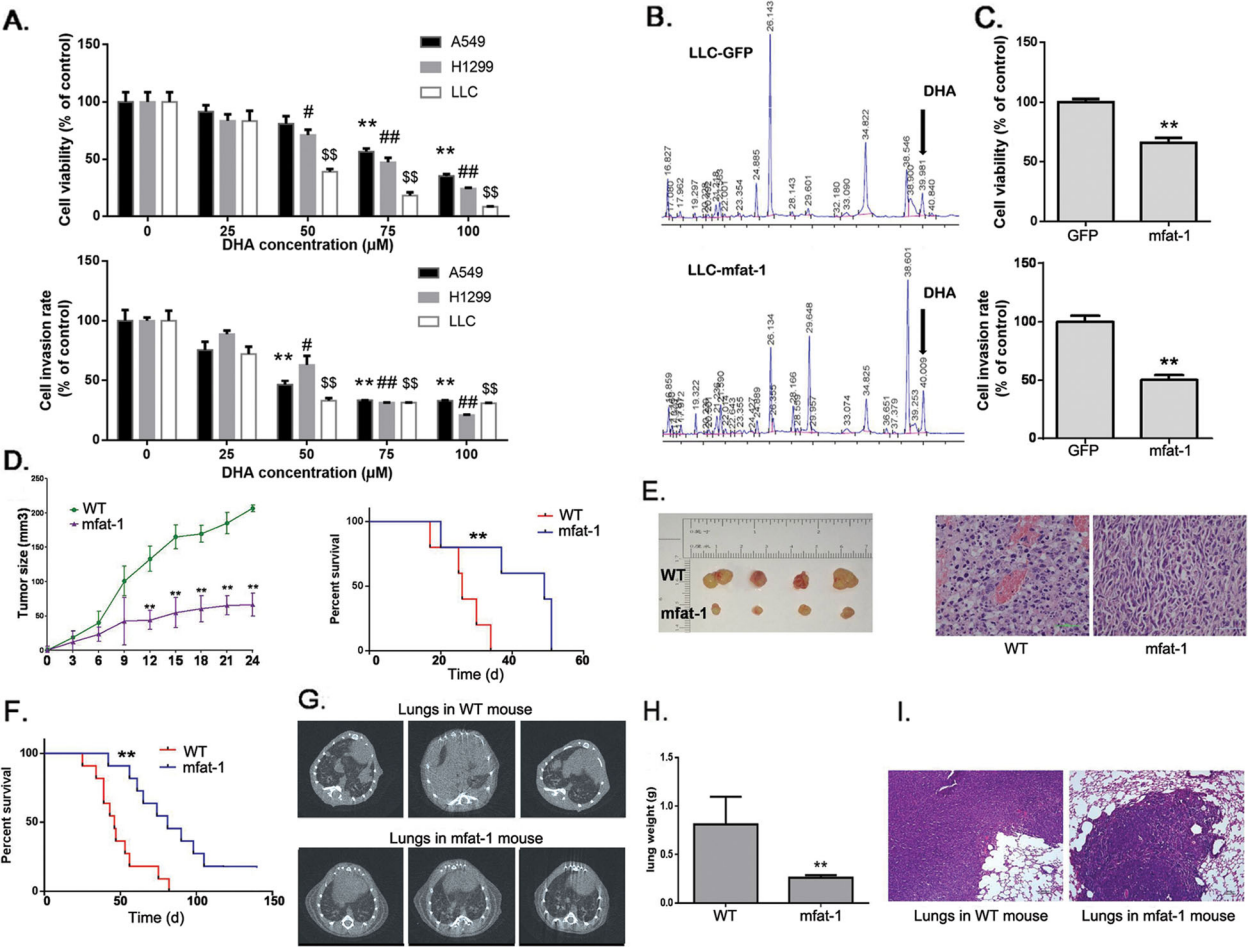
Exogeneous and endogenous DHA inhibit cell growth, invasion and metastasis of NSCLC cells. **a**. A549, H1299 or LLC cells were treated with various concentration of DHA, and were subjected to cell growth and invasion assays (*n* = 4). *, ^#^, ^$^*P* < 0.05, **, ^##^, ^$$^*P* < 0.01 compared to 0 μM DHA group. **b**. LLC cells were transduced with lentivirus carrying the mfat-1 gene, or GFP as a negative control. Components were identified by comparison of retention times with those of PUFA analytical standard. The amount of DHA is expressed as a percentage of all fatty acid peaks. **c**. LLC-GFP and LLC-mfat-1 were subjected to cell growth and invasion assays (*n* = 4). **d**. LLC cells were subcutaneously injected into female WT or mfat-1 mice (*n* = 8). Survival of tumor-bearing mice (Right). **e**. Representative images of implantation tumors (Left). HE staining of tumors (400×) (Right). **f**. LLC cells were injected into tail veins of female WT or mfat-1 mice (*n* = 8). Survival of tumor-bearing mice. **g**. CT images of metastatic lung tumors. **h**. Mass of excised tumors. **i**. HE staining of tumors (100×)

Human lung cancer cell lines, A549 and H1299, and a mouse lung cancer cell line, LLC, were applied to evaluate the impact of DHA on cellular growth and invasion. DHA decreased cell viability in all the above lung cancer cells in a dose-dependent manner. Treatment of the cells with 100 μM DHA for 24 h caused a loss of viability up to 70–90%. Meanwhile, A549, H1299 and LLC cells exhibited a similar reduction of cell invasion following the DHA treatment (Fig. 1a). Then we added another ω-3 PUFAs, EPA, to lung cancer cells, and investigated its effects on cell viability and invasion. However, EPA treatment showed mild antitumor effects in A549 and H1299 (Additional file 3: Figure S2).

As a further step of demonstrating the beneficial effect of DHA against lung cancer development, we infected LLC cells with a lentiviral vector carrying the mfat-1 gene (termed “LLC-mfat-1”cells) whose encoded enzyme could endogenously convert ω-6 PUFAs to ω-3 PUFAs (including DHA and EPA) [20]. A control cell line expressing green fluorescent protein (GFP) was termed LLC-GFP. The expression of mfat-1 allowed more than 50% increase in endogenous DHA level (from 1.61 to 2.46%, *P* < 0.05) (Fig. 1b), which coincided with a significantly suppressed cell viability and invasion (Fig. 1c).

The inhibition of lung cancer growth by endogenously elevated DHA was further shown in the cancer model grafts derived from subcutaneously implanted LLC cells in both the wild-type (WT) and mfat-1 transgenic mice. Elevation of tissue contents of ω-3 PUFAs including DHA sharply slowed the growth of the implanted LLC tumor as measured by tumor volumes, and prolonged the survival of tumor-bearing mice (Fig. 1d, e). In metastatic models, the WT and mfat-1 mice were injected with LLC cells through tail veins. During the ensuing observation period, the mfat-1 mice displayed significantly longer survival time than the control WT mice. After euthanasia, we found endogenous DHA caused lower lungs weight and partial tumor regression (Fig. 1f-i). In the WT mice, the metastatic tumors were identified not only in lungs, but also in submaxillary gland, bone, adrenal gland, and buttock, while the metastatic tumor could only be found in the lungs of the mfat-1 mice, but at much reduced weight and size (Fig. 1h-i, Additional file 4: Figure S3). Thus, DHA, provided exogenously or endogenously, could reduce tumor cell growth, invasion and metastasis in vitro and in vivo.

### DHA-derived eicosanoid, RvD1, is involved in DHA-mediated suppression of cancer cell growth and invasion

**Fig. 2:**
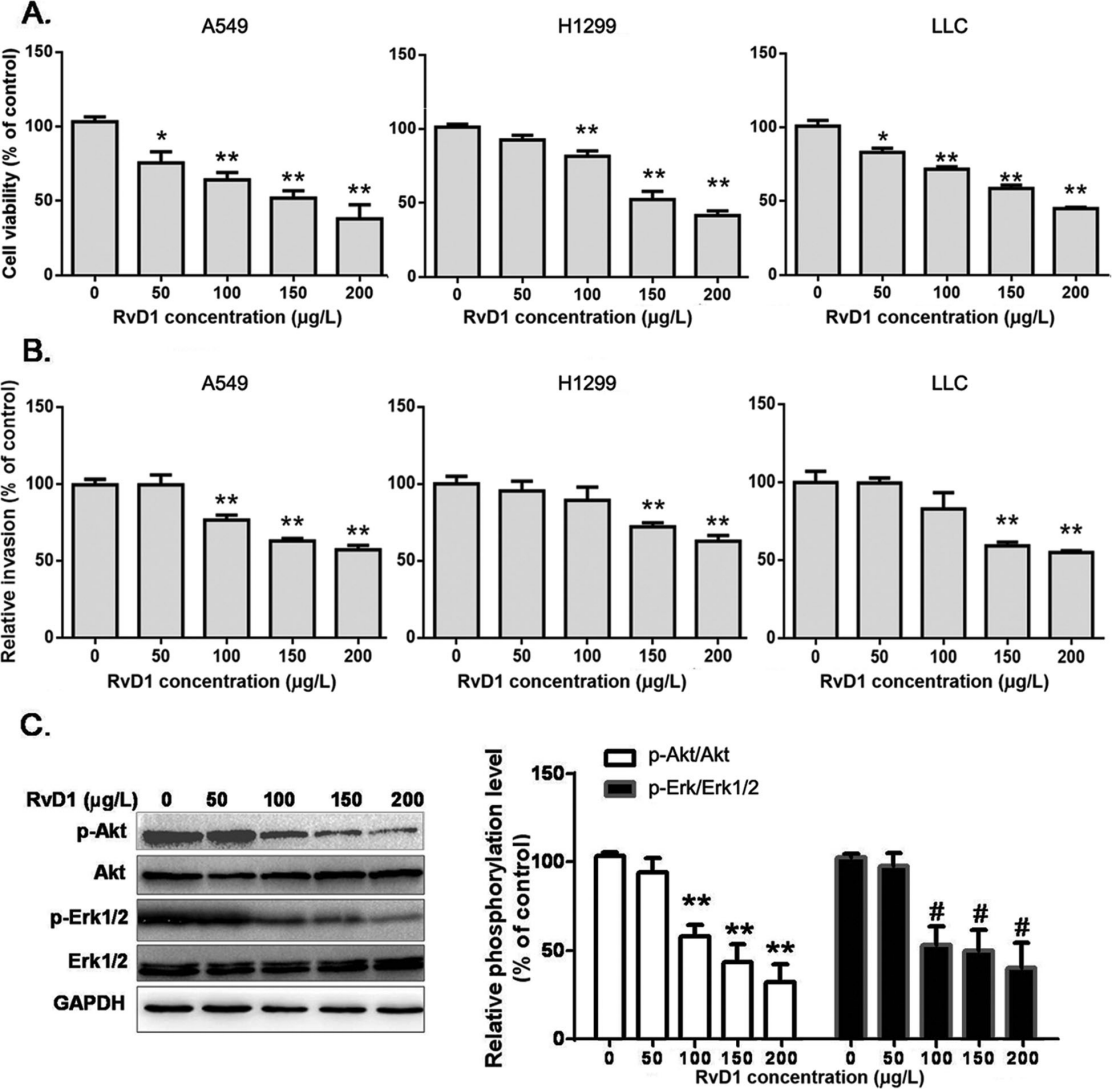
RvD1 inhibits cell growth and invasion of NSCLC cells. **a**. A549 cells, H1299 and LLC cells were treated with various concentrations of RvD1, and were subjected to cell growth (*n* = 4). **b**. A549 cells, H1299 and LLC cells were treated with various concentrations of RvD1, and were subjected to invasion assays (*n* = 3). **c**. A549 cells were treated with various concentrations of RvD1. After 48 h, Akt and Erk1/2 phosphorylation was evaluated by Western blot using total Akt and Erk1/2 as internal controls. Data are presented as the mean ± SEM from three independent experiments and were normalized to the control group. **, ^##^*P* < 0.01 compared to corresponding control groups

To understand the impact of DHA on NSCLC growth and metastasis, we analyzed some of the eicosanoid metabolites induced by DHA treatment. As one would expect, 10 of the 15 identified DHA-derived metabolites through LC-MS/MS assays were increased in LLC cells following DHA treatment or the expression of mfat-1 (Table 1), chief among which, RvD1, was sharply elevated compared with the control group. RvD1 is essentially undetectable in the cultured lung cancer cells, but sharply increased in cells with either exogenous or endogenous DHA treatment. With this knowledge, we evaluated the effects of RvD1 in A549, H1299 and LLC cells. RvD1 decreased cell viability in A549, H1299 and LLC cells in a dose-dependent manner (Fig. 2a, b). At the dose of 200 μg/L, RvD1 treatment for 24 h caused a loss of viability 60%. All the lung cancer cells exhibited a similar reduction in cell invasion following RvD1 treatment (Fig. 2a, b). Furthermore, addition of RvD1 to A549 cells had a profound effect on Akt and Erk1/2 phosphorylation. These results suggested that RvD1 inhibited cell growth and invasion in NSCLC in vitro (Fig. 2c).

**Table 1:** LLC cell DHA metabolite concentration (ng/10^5^ cell) determined by LC-MS/MS

Oxylipin	LLC-GFP	LLC-GFP treat with DHA	LLC-mfat-1
10-HDoHE	0.34 ± 0.20	1072.73 ± 245.41^**^	736.22 ± 205.21^**^
10S,17S-DiHDoHE	NP	8.75 ± 3.54^**^	NP
11-HDoHE	NP	1300.73 ± 254.98^**^	NP
13-HDoHE	0.56 ± 0.39	734.25 ± 261.61^**^	575.98 ± 272.13^**^
14-HDoHE	0.46 ± 0.32	454.98 ± 197.20^*^	179.32 ± 92.18^**^
17-HDoHE	1.31 ± 0.74	1674.18 ± 492.10^**^	661.26 ± 133.4^**^
20-HDoHE	1.00 ± 0.63	2567.3 ± 438.31^**^	822.19 ± 212.35^**^
4-HDoHE	2.93 ± 1.10	5175.27 ± 475.64^**^	1634.9 ± 773.01^**^
7-HDoHE	0.59 ± 0.34	2020.17 ± 293.62^**^	536.16 ± 124.03^**^
8-HDoHE	2.57 ± 1.65	5847.24 ± 425.41^**^	1298.76 ± 235.18^**^
Maresin	NP	NP	NP
RvD1	NP	2.18 ± 0.59^**^	1.07 ± 0.32^**^
RvD2	NP	NP	NP
16,17-EDP	0.76 ± 0.55	1079.19 ± 333.12^**^	343.23 ± 83.43^**^
19,20-EDP	1.43 ± 0.42	3858.48 ± 419.96^**^	1131.8 ± 241.57^**^

^*^P < 0.05, ^**^P < 0.01 compared with LLC-GFP. Data are mean **±** SD. *NP* No peak, *CYP* Cytochrome P450 enzymes, *COX* Cyclooxygenase, *LOX* Lipoxygenase, *PG* Prostaglandin

### RvD1 suppresses lung cancer cell growth and invasion by increasing miR-138-5p expression

**Fig. 3:**
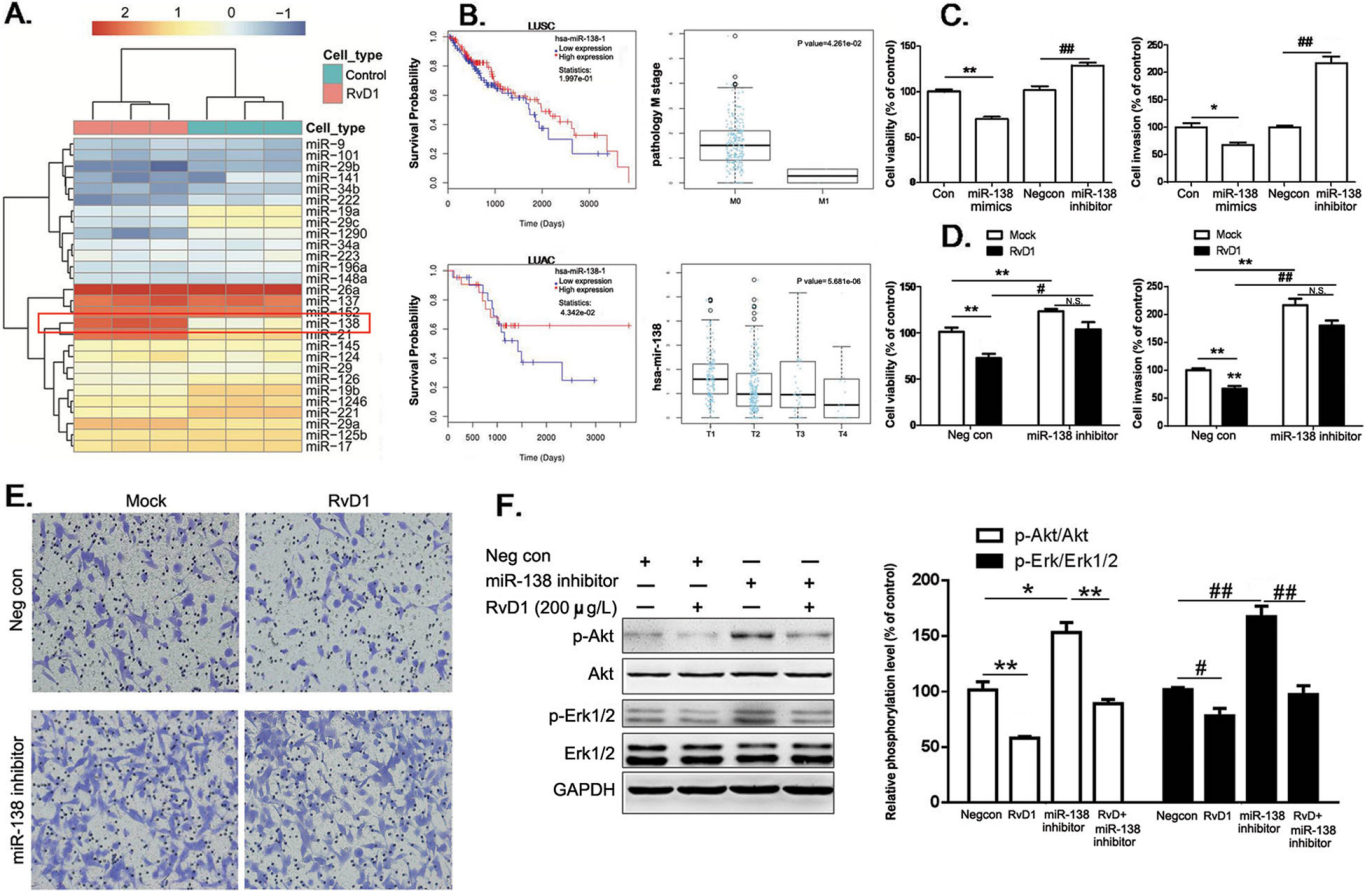
miR-138-5p is involved in RvD1-mediated suppression of cell growth and invasion. **a**. Heat map diagram with supervised hierarchical clustering of miRNAs expressed by RvD1-treated A549 cells. The miRNA clustering tree is shown on the left, and the sample clustering tree appears at the top (control and RvD1 200 μg/L group). The color scale in the map illustrates the relative expression level of a miRNA across all samples: red represents an expression level above the mean, and blue represents expression lower than the mean. **b**. miR-138 miRNA expression data were downloaded from the TCGA database containing 344 LUSC and 327 LUAC cancer tissues. The overall survival (OS) of patients with tumors expressing high vs. low miR-138 was analyzed by the Cox regression test. The relationship between miR-138-5p and tumor extent (T1-T4) or distant metastasis (M0-M1) was analyzed by Kruskal-Wallis test. **c**. A549 cells were transfected with control mimics (Con), miR-138-5p mimics, control anti-sense RNA inhibitor (Negcon), or miR-138 inhibitor, and then subjected to cell growth and invasion assays. *P < 0.05, **P < 0.01 compared to Con group; ^##^*P* < 0.01 compared to Negcon group. **d**. A549 cells were transfected with Negcon or miR-138 inhibitor, and subjected to cell growth and invasion assays (*n* = 4). **P < 0.01 compared to Negcon group; ^#^*P* < 0.05, ^##^*P* < 0.01 compared to NegCon+RvD1 group. N.S. no significance. There is no significant difference in miR-138 inhibitor-treated groups with or without RvD1 treatment (*P* = 0.052). **e**. A549 cells were treated as above and subjected to invasion assays. There is no significant difference in miR-138 inhibitor-treated groups with or without RvD1 treatment (*P* = 0.077). The transwell units were stained and high power views (200×) were selected randomly from each sample in a blind manner. **f**. A549 cells were treated as above. Akt and Erk1/2 phosphorylation were evaluated by Western blot analysis using total Akt and Erk1/2 as internal controls. *P < 0.05, **P < 0.01 for p-Akt compared to Con group; ^#^*P* < 0.05, ^##^*P* < 0.01 for p-Erk1/2 compared to Con group

Previous studies have shown that RvD1 regulates the expression of several miRNAs and plays important roles in the regulation of inflammation [25]. Using a human miRNA microarray, we found that the expression levels of many miRNA species were affected by the addition of RvD1 in A549 cells. Among which, the expression of miR-138-5p was sharply elevated with more than a 60-fold increase than that in control cells (Fig. 3a). These results made us investigate miR-138 expression in the cancer specimens derived from LUSC and LUAD in the TCGA miRNA expression database, which covers the patients with different lymphatic metastasis or hematogenous metastasis. In LUSC patients, miR-138 expression was significantly reduced in cases with distant metastasis, and high expression of miR-138 showed longer survival tendency than those with low miR-138 expression (Fig. 3). In LUAD patients, high expression of miR-138 was significantly correlated with longer survival than those with low miR-138 expression. In addition, miR-138 expression was significantly reduced in cases with tumor extents (Fig. 3b).

We found that the mimics of miR-138-5p, after applied to the cultured cells, could decrease cell viability and invasion in A549 cells (Fig. 3c). In contrast, transfection of miR-138-5p inhibitor not only increased cell viability and invasion but, more importantly, largely neutralized RvD1-mediated suppression in cell viability and invasion (Fig. 3c-e). These data indicated that induction of miR-138-5p expression was an important contributing mechanism for the suppressive effect of RvD1 on lung cancer cell growth and invasion. However, addition of miR-138 failed to reverse the inhibitory effect of RvD1 on Akt or Erk1/2 kinases, suggesting the parallel regulation of these two molecular events that can be both important for DHA inhibitory effects on lung cancer growth (Fig. 3f).

### MiR-138-5p directly targets FOXC1 expression to reduce lung cancer cell growth and invasion

**Fig. 4:**
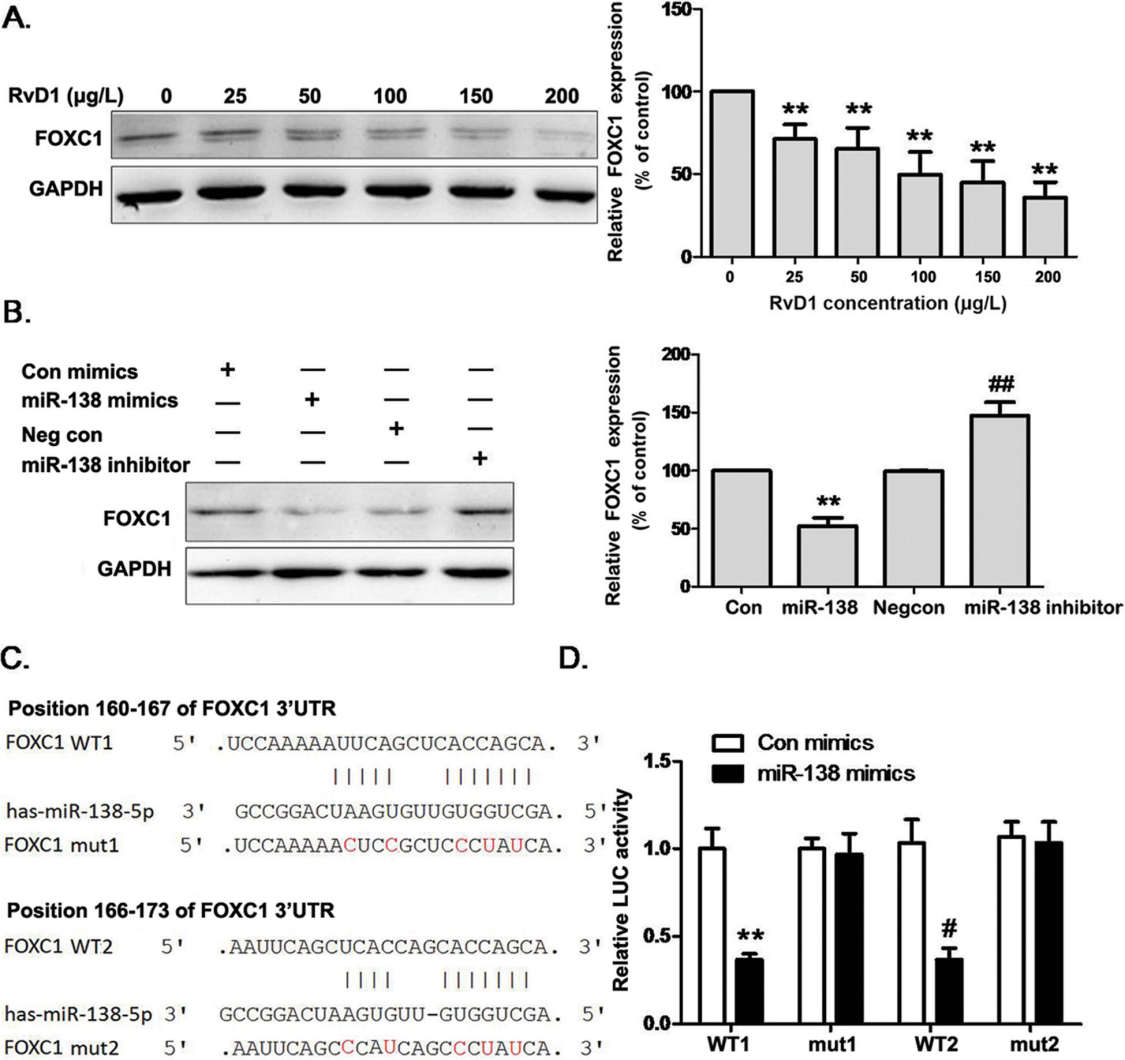
FOXC1 is a direct target of miR-138. **a**. A549 cells were treated with RvD1 and the expression level of FOXC1 was analyzed by Western blot, using GAPDH as an endogenous control. **P < 0.01 compared to 0 μg/L RvD1 group. **b**. A549 cells were transfected with Con, miR-138-5p mimics, Negcon, or miR-138 inhibitor. FOXC1 expression was analyzed by Western blot. **P < 0.01 compared to Con group; ^##^*P* < 0.01compared to NegCon group. **c**. Putative seed-matching sites (in bold) or mutant sites (red) between miR-138-5p and the 3′-UTR of FOXC1 were analyzed by TargetScan. **d**. The wild type (WT1 and WT2) and mutant (mut1 and mut2) binding site sequences of miR-138-5p in the 3′ UTR of FOXC1 were cloned into luciferase reporter vectors. A549 cells were co-transfected with the reporter vectors, renilla luciferase vector, and miR-138-5p mimics or Con mimics. After 24 h, the relative luciferase activities were analyzed and data were normalized as the ratio of the Con mimics + WT group. Data are presented as the mean ± SEM from three independent experiments. **P < 0.01 compared to corresponding group

Bioinformatics analysis suggests that Forkhead Box C1 (FOXC1) is potentially a target of miR-138-5p (www.targetscan.org/). Indeed, Western blot assays showed that the protein level of FOXC1 was decreased in a dose-dependent manner by RvD1 treatment in A549 cells (Fig. 4a); meanwhile, miR-138-5p mimics reduced FOXC1 expression. In contrast, an inhibitor of miR-138-5p, after transfected into the same cell line, significantly increased FOXC1 expression (Fig. 4b). To further confirm these findings, luciferase reporter constructs were made in which the putative binding site in the 3′-UTR regions of FOXC1 (wt) or the same binding site except with three nucleotide substitutes (mut1 and mut2, indicated in Fig. 4c). Transfection of miR-138-5p inhibited the expression of the reporter gene containing the WT, but not the mut1 or mut2, of the 3′-UTR region of FOXC1 (Fig. 4d), demonstrating that miR-138-5p can specifically target FOXC1 3′-UTR regions by binding to the target sequences.

**Fig. 5:**
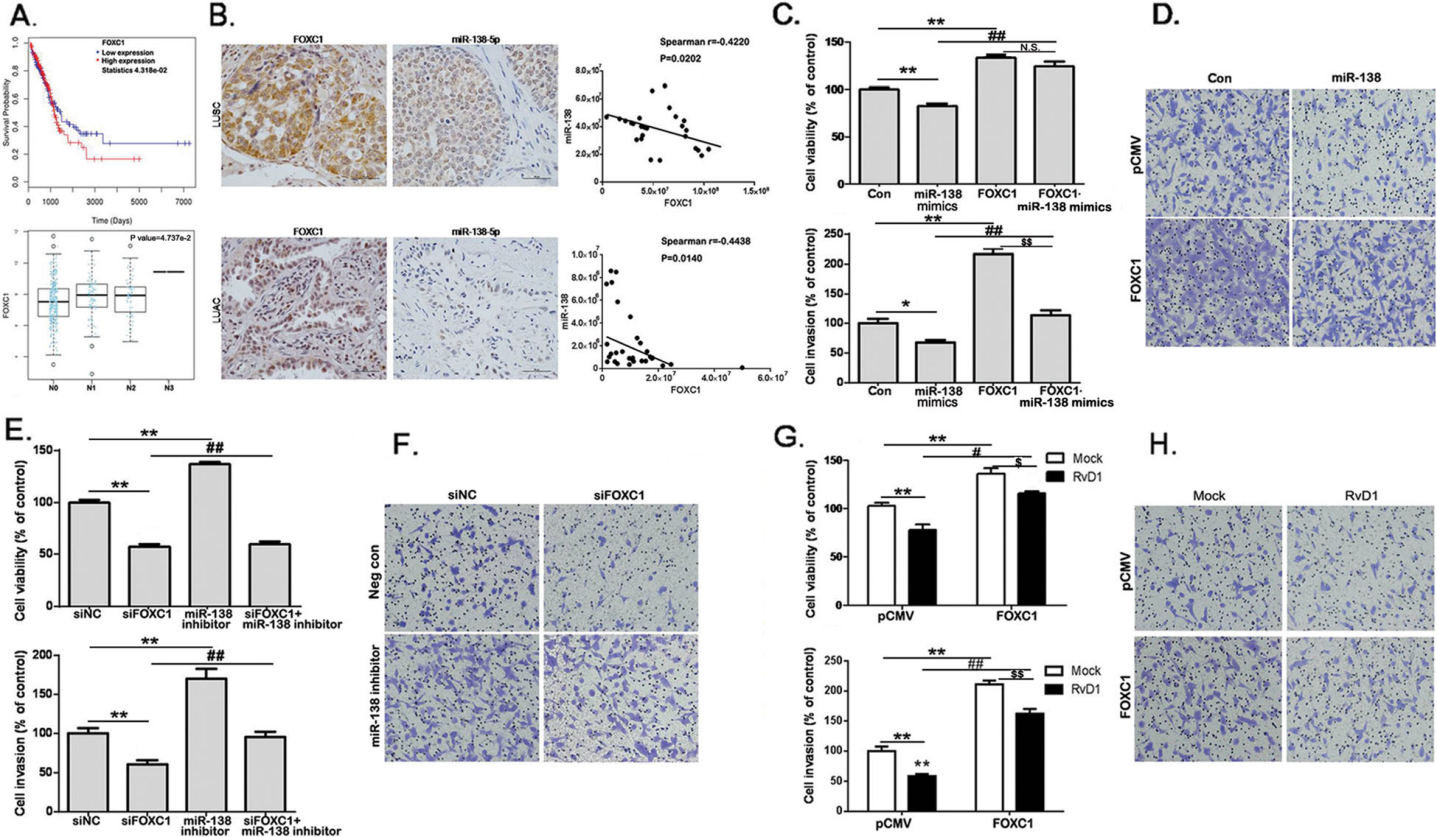
FOXC1 reverses RvD1/miR-138-mediated suppression of NSCLC cell growth and invasion**. a**. FOXC1 mRNA expression data were downloaded from the TCGA database containing 439 LUAC cancer tissues. The overall survival (OS) of patients with tumor expressing high vs. low FOXC1 was analyzed by the COX regression test. The relationship between FOXC1 and lymph node metastasis (N0-N3) was analyzed by Kruskal-Wallis test. **b**. Representative immunohistochemical images of LUSC and LUAC tissues stained with an anti-FOXC1 antibody. Representative in situ hybridization images of lung cancer tissues stained with a miR-138-5p antibody. Scale bars = 50 μm (40×). **c**. A549 cells were transfected with Con, miR-138-5p mimics, pCMV, or FOXC1-pCMV, and then subjected to cell growth and invasion assays. *P < 0.05, **P < 0.01 compared to Con group; ^##^*P* < 0.01 compared to miR-138 group; ^$$^*P* < 0.01, compared to FOXC1-overexpressed group. **d**. A549 cells were transfected with above, and then subjected to cell invasion assays. The transwell units were stained and photographed. High power views (200×) were selected randomly from each sample in a blind manner. **e**. A549 cells were transfected with Negcon, miR-138 inhibitor, siNC, or siFOXC1, and then subjected to cell growth assays (*n* = 4). **P < 0.01 compared to siNC group; ^##^*P* < 0.01 compared to miR-138 inhibitor group. **f**. A549 cells were transfected with above reagents, and subjected to cell invasion assays, and high power views (200×) were selected. **g**. A549 cells were transfected with pCMV or FOXC1-pCMV, and subjected to cell growth assays (*n* = 4). **P < 0.01 compared to pCMV group; ^#^*P* < 0.05, ^##^*P* < 0.01 compared to pCMV+RvD1 200 μg/L group. ^$^*P* < 0.05, ^$$^*P* < 0.01 compared to FOXC1-overexpression group. **h**. A549 cells were treated as above and subjected to the invasion assays. The transwell units were stained and high power views (200×) were selected randomly from each sample in a blind manner

Since FOXC1 is highly expressed in many cancers and contributes to cancer cell growth and invasion [26], we also looked into FOXC1 expression in LUSC and LUAD cancer specimens with different tumor extents and lymphatic metastasis in TCGA mRNA expression database. High expression of FOXC1 correlated with poor survival of the patients with LUAD when compared to the patients with low expression of FOXC1 (Fig. 5a). FOXC1 expression was also high in lymphatic metastatic tissues (Fig. 5). Analysis of FOXC1 expression in TCGA database covering LUSC cases showed a similar tendency correlation between the expression of FOXC1 and survival of patients (Additional file 5: Figure S4). In 60 lung cancer cases (30 LUSC and 30 LUAD), in situ hybridization assays were used to detect miR-138-5p expression, and immunohistochemistry assays were used to detect FOXC1 expression. Figure 5b showed a strong negative correlation existed between the expression levels of miR-138-5p and FOXC1 in both LUAD and LUSC tissues.

To establish the cross regulation of FOXC1 activity by miR-138-5p, A549 cells were transfected with miR-138-5p mimics with or without co-transfection of a CMV promoter-driven FOXC1 construct. Overexpression of FOXC1 not only increased cell viability and invasion, but partially blocked the suppressive effects of miR-138-5p on cell invasion (Fig. 5c, d). In a parallel experimental scheme, a miR-138-5p inhibitor was transfected in A549 cells with or without the co-transfection of FOXC1 siRNA. Although the miR-138-5p inhibitor stimulated cell growth and invasion as one would expect, the addition of FOXC1 siRNA neutralized all of these effects of this miR-138-5p inhibitor (Fig. 5e, f). Of particular interests to this study, overexpression of FOXC1 in A549 cells significantly promoted cancer cell growth and invasion. Although RvD1 treatment led to 25% reduction in cell viability, overexpression of FOXC1 significantly attenuated the effect to a 15% loss of cell viability ability. In cell invasion assay, although RvD1 treatment led to 42% reduction in lung cancer cell invasion, overexpression of FOXC1 significantly attenuated the effect to a 23% loss of cell invasive ability. Our data suggested that despite the weak suppressive effects of RvD1 on cell viability and invasion, it was at least partially reversed by overexpression of FOXC1. (Fig. 5g, h). Taken together, these data indicated that miR-138-5p directly targeted FOXC1 to reduce cancer cell growth and invasion.

### Endogenous DHA enhances miR-138-5p expression and reduced FOXC1 expression in vivo

**Fig. 6:**
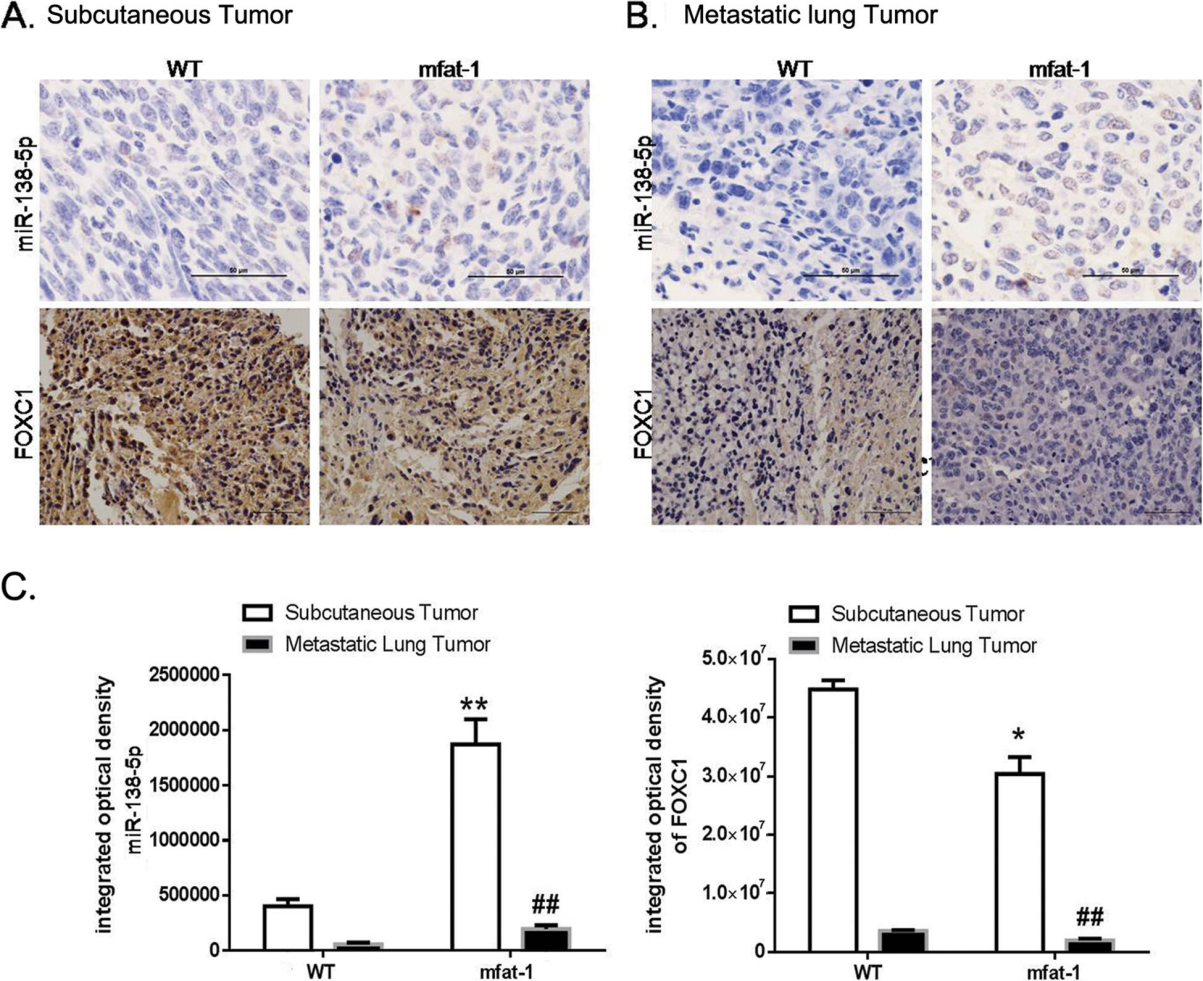
Endogenous DHA increases miR-138-5p expression and decreases FOXC1 expression in vivo. LLC cells were injected subcutaneously into WT or mfat-1 mice (**a**), or injected into the tail vein of WT or mfat-1 mice (**b**). Representative in situ hybridization images of cancer tissues stained with miR-138-5p RNAscope probe (upper panel). Representative immunohistochemical images of LUSC tissues and LUAC tissues stained with an anti-FOXC1 antibody (lower panel). Scale bars = 50 μm (40×). **c**. The integrated optical density level was determined using Image Pro Plus software. Data are presented as the mean ± SEM from 4 different samples. *P < 0.05, **P < 0.01 for miR-138-5p expression compared to WT group; ^##^*P* < 0.01 for FOXC1 expression compared to WT group

We further established how elevated DHA suppressed tumor growth and metastasis through the regulation of miR-138-5p and FOXC1 in vivo. In situ hybridization and immunohistochemistry assays in the subcutaneous LLC grafts revealed that the level of miR-138-5p level was sharply enhanced, but the expression of FOXC1 significantly attenuated, in the mfat-1 transgenic mice (Fig. 6a, c). Similar findings were also observed after analyzing the metastatic lung tumor mass following the intravenous injection of LLC cells (Fig. 6b, c). Elevation of tissue DHA in vivo, therefore, would also cause the same changes in miR-138-5p and FOXC1 as those observed in the cultured lung cancer cells.

## Discussion

Although the current combinatorial immunotherapy has become one of the primary treatment modality for NSCLC, the response rates are still below 30% [27]. Clinical application of ω-3 PUFA dietary supplement, particularly EPA plus DHA, significantly increased the response rates to chemotherapy and prolonged the survival of cancer patients without affecting the toxicity profiles [28]. A prospective cohort study also showed that DHA intake was inversely associated with lung cancer risk, particularly among never-smokers and adenocarcinoma patients [29]. DHA treatment, both in vivo and in vitro, not only suppressed cancer cell proliferation and migration [9], but also increased cancer cell sensitivity to chemo-drugs [30]. Despite such favorable clinical and cellular evidence, the underlying mechanisms of DHA’s actions remain unclear. The current study addressed this issue by revealing that RvD1, one of DHA-initiated eicosanoid metabolites [10], played a critical role in mediating the effects of DHA by decreasing lung cancer cell growth and invasion. Importantly, we found that RvD1 exerted these effects by modulating the miR-138-5p/FOXC1 pathway in lung cancer cells.

To this day, little is known about the effects of RvD1 on NSCLC cell growth and metastasis, let alone the underlying mechanisms. We shed some light on this issue by revealing significantly elevated miR-138-5p expression following RvD1 treatment of A549 cells in a miRNA microarray analysis. MiR-138-5p has been reported as a tumor suppressor in several types of cancer such as multiple myeloma and ovarian cancer by targeting EZH2 and survivin [17, 18, 31, 32]. Overexpression of miR-138-5p inhibited cancer cell proliferation and invasion while enhancing drug sensitivity [17, 32]. In clinical biopsy samples as well as in TCGA databases analysis, we found that decreased expression of miR-138-5p was frequently observed in metastatic lung cancer tissues, while high miR-138-5p expression was significantly correlated with better prognosis. Overexpression of miR-138-5p reduced, while miR-138-5p inhibitor promoted, lung cell growth and invasion. Importantly, miR-138-5p inhibitor completely abrogated the anti-tumor effects of RvD1. To our knowledge, this is the first report to establish the mechanistic linkage between miR-138-5p and an eicosanoid metabolite of DHA.

FOXC1 has been highlighted as an important transcriptional regulator involved in diverse tumorigenic processes, such as proliferation, invasion, and angiogenesis [26, 33–35] with high FOXC1 expression associated with poor prognosis in cancer patients [36, 37]. Specifically, in lung cancer, FOXC1 plays a critical role in tumor microenvironment-promoted cancer progression [38, 39]. Our study has demonstrated FOXC1 as the direct target of miR-138-5p expression. FOXC1 overexpression reversed the suppressive effects of miR-138-5p or RvD1 treatment on lung cell growth and invasion. The elevated miR-138-5p expression induced by DHA treatment could decrease FOXC1 expression in tumor allografts.

## Conclusion

These results built a mechanism linking RvD1, miR-138-5p, and FOXC1 as the signaling pathway that mediated the suppression of NSCLC growth and metastasis by DHA. The revelation of such new mechanism may thus offer a novel treatment target for this malignant disease.

## Supplementary information

**Additional file 1: Table S1.** Analysis of blood ω-6 and ω-3 PUFA compositions in mfat-1 transgenic mice. All data presented as means ± SD. *n* = 6. ^*^P < 0.05 and ^**^P < 0.01 when compared with the WT group.

**Additional file 2: Figure S1.** A. The levels of mfat-1 expression were determined by RT-PCR in LLC cells and transgenic mice. B. Levels of overexpression or inhibition of miR-138 were determined by qRT-PCR. All data presented as means ± SEM. *n* = 3. **P < 0.01 compared to the corresponding control groups. C. The levels of overexpression or inhibition of FOXC1 molecules were determined by Western blot.

**Additional file 3: Figure S2.** A. A549 or H1299 cells were treated with various concentration of EPA, and were subjected to cell growth assays. B. A549 or H1299 cells were treated with various concentration of EPA, and were subjected to cell invasion assays. Data are presented as the mean ± SEM (*n* = 4). *P < 0.05, **P < 0.01 compared to 0 μM EPA group.

**Additional file 4: Figure S3.** A. LLC cells were injected into the tail vein of female WT or mfat-1 mice (*n* = 4). Forty days after injection, the mice were euthanized; the lungs were removed and representative images were displayed. B. Metastatic masses were found in other organs in addition to lungs in WT mice. HE-stained tissues were photographed using a Leica microscope. High power views (200×) were selected.

**Additional file 5: Figure S4.** FOXC1 mRNA expression data were downloaded from the TCGA database containing 479 LUSC cancer tissues. Overall survival (OS) of patients in relation to FOXC1 expression status (high vs. low) was analyzed by the COX regression test. The relationship between FOXC1 expression and lymph node metastasis (N0-N3) was analyzed by Kruskal-Wallis test.

## Data Availability

The datasets supporting the conclusions of this article are included in this published article (and its supplementary information files).

## Abbreviations

Akt: Protein kinase B
DHA: Docosahexaenoic acid
EPA: Eicosapentaenoic acid
Erk: Extracellular regulated kinase
FOXC1: Forkhead Box C1
LC-MS: Liquid chromatography separation with tandem mass spectrometry
LUAC: Lung adenocarcinoma
LUSC: Lung squamous cell carcinoma
MiRNAs: MicroRNAs
NSCLC: Non small cell lung cancer
PUFAs: Polyunsaturated fatty acids
RvD1: Resolvin D1
