# Supplementary material for: Inhibition of lung cancer growth and metastasis by DHA and its metabolite, RvD1, through miR-138-5p/FOXC1 pathway
Source: J Exp Clin Cancer Res. 2019 Nov 29;38:479. doi: 10.1186/s13046-019-1478-3 (PMC6884860; doi:10.1186/s13046-019-1478-3)
Supplement: Supplementary file 1 — Additional file 1: Table S1. Analysis of blood ω-6 and ω-3 PUFA compositions in mfat-1 transgenic mice. All data presented as means ± SD. n = 6. *P < 0.05 and **P < 0.01 when compared with the WT group. [file 13046_2019_1478_MOESM1_ESM.docx]

**Table S1. Analysis of ω-6 and ω-3 PUFA composition.** Abbreviations: ALA, α lipoic acid; DPA, docosapentaenoic acid; LA, linoleic acid. The composition of ω-6 and ω-3 PUFAs in the mice blood was analyzed using standard protocols (see Materials and Methods). Each species is expressed as a percentage of all fatty acid peaks, ie, the distribution areas of different ω-3 or ω-6 PUFAs peaks divided by the total peak areas of all detectable saturated and unsaturated free fatty acids (from the same sample) resolved from the gas chromatography column. All data presented are means ± SD. n = 6. ^*^*P* < 0.05 and ^**^*P* < 0.01 when the mfat-1 group compared with WT group.

| **PUFA Species, %** | **WT** | **mfat-1** |
| --- | --- | --- |
| **ALA(C18:3 ω-3)** | **0.29 ± 0.03** | **0.57 ± 0.07^*^** |
| **EPA(C20:5, ω-3)** | **0.75 ± 0.03** | **2.10 ± 0.35^**^** |
| **DPA(C22:5, ω-3)** | **0.77 ± 0.14** | **1.19 ± 0.11^*^** |
| **DHA(C22:6, ω-3)** | **5.17 ± 0.44** | **6.36 ± 0.15^*^** |
| **ω-3, total** | **6.99 ± 0.47** | **10.21 ± 0.44^**^** |
| **LA(C18:2, ω-6)** | **17.57 ± 1.28** | **15.27 ± 0.30^*^** |
| **AA(C20:4, ω-6)** | **11.36 ± 1.06** | **7.21 ± 1.06^**^** |
| **ω-6, total** | **29.75 ± 1.70** | **23.16 ± 0.95^**^** |
| **ω-6/ω-3 ratio** | **4.26 ± 0.25** | **2.27 ± 0.18^**^** |
