# Supplementary figures and images for: Inhibition of lung cancer growth and metastasis by DHA and its metabolite, RvD1, through miR-138-5p/FOXC1 pathway
Source: J Exp Clin Cancer Res. 2019 Nov 29;38:479. doi: 10.1186/s13046-019-1478-3 (PMC6884860; doi:10.1186/s13046-019-1478-3)

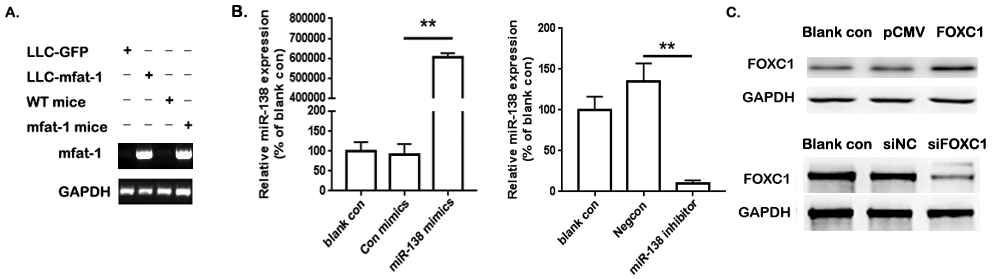

Supplement: Supplementary file 2 — Additional file 2: Figure S1. A. The levels of mfat-1 expression were determined by RT-PCR in LLC cells and transgenic mice. B. Levels of overexpression or inhibition of miR-138 were determined by qRT-PCR. All data presented as means ± SEM. n = 3. **P < 0.01 compared to the corresponding control groups. C. The levels of overexpression or inhibition of FOXC1 molecules were determined by Western blot. [file 13046_2019_1478_MOESM2_ESM.tif]

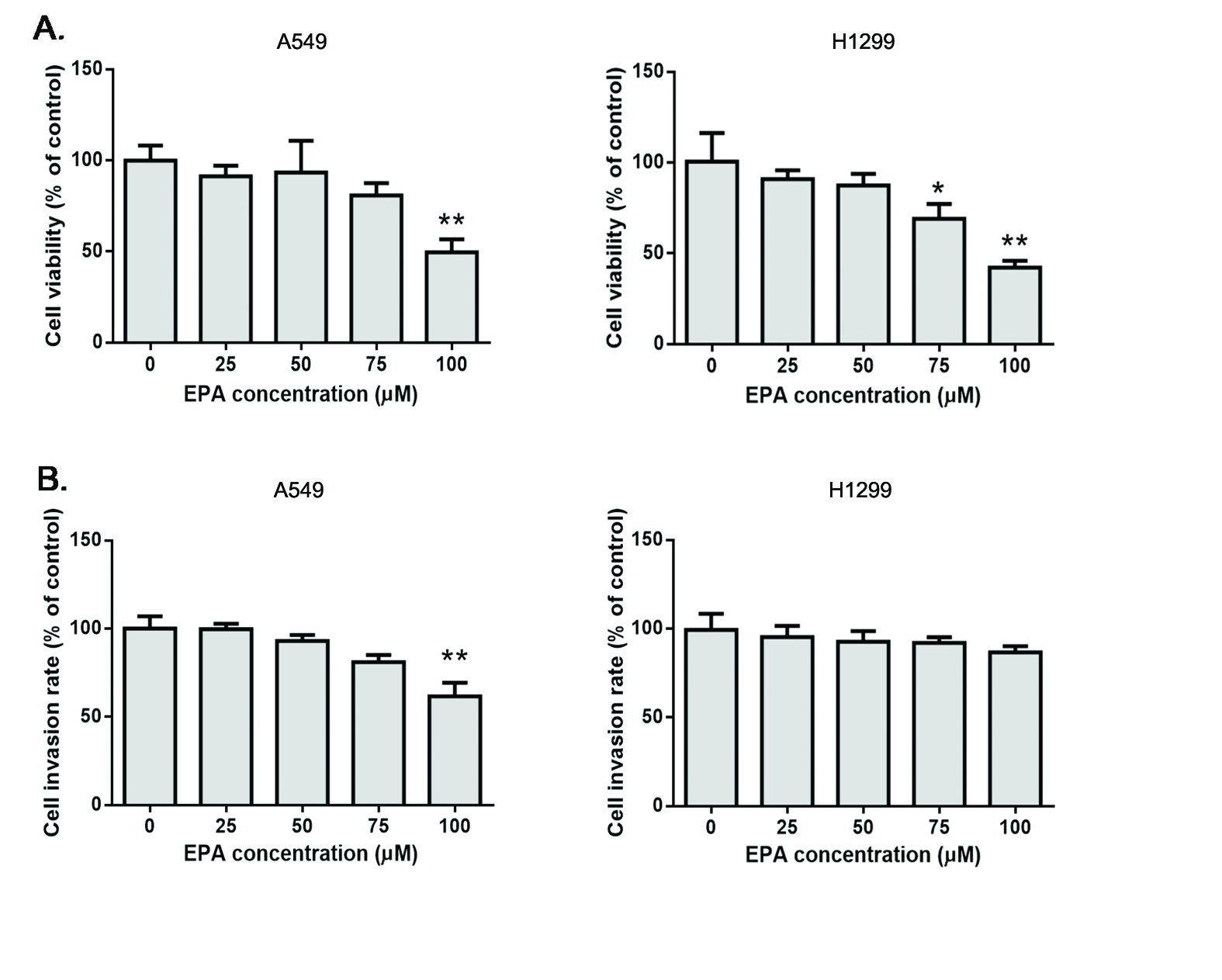

Supplement: Supplementary file 3 — Additional file 3: Figure S2. A. A549 or H1299 cells were treated with various concentration of EPA, and were subjected to cell growth assays. B. A549 or H1299 cells were treated with various concentration of EPA, and were subjected to cell invasion assays. Data are presented as the mean ± SEM (n = 4). *P < 0.05, **P < 0.01 compared to 0 μM EPA group. [file 13046_2019_1478_MOESM3_ESM.tif]

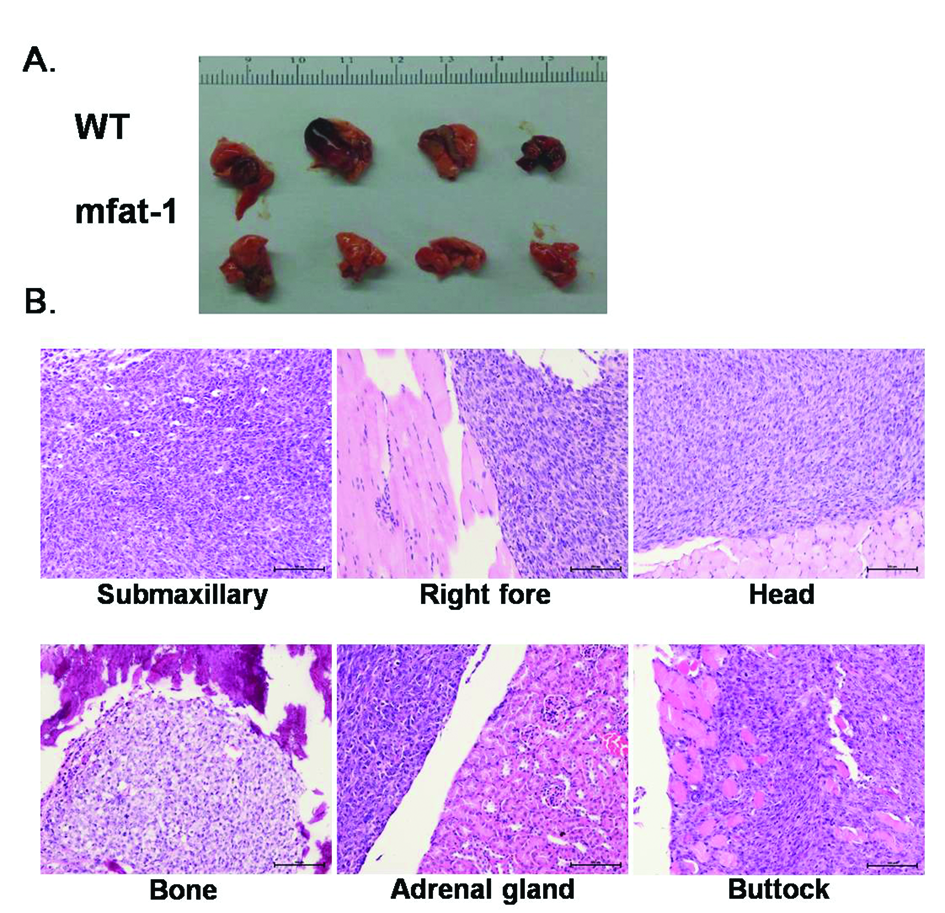

Supplement: Supplementary file 4 — Additional file 4: Figure S3. A. LLC cells were injected into the tail vein of female WT or mfat-1 mice (n = 4). Forty days after injection, the mice were euthanized; the lungs were removed and representative images were displayed. B. Metastatic masses were found in other organs in addition to lungs in WT mice. HE-stained tissues were photographed using a Leica microscope. High power views (200×) were selected. [file 13046_2019_1478_MOESM4_ESM.tif]

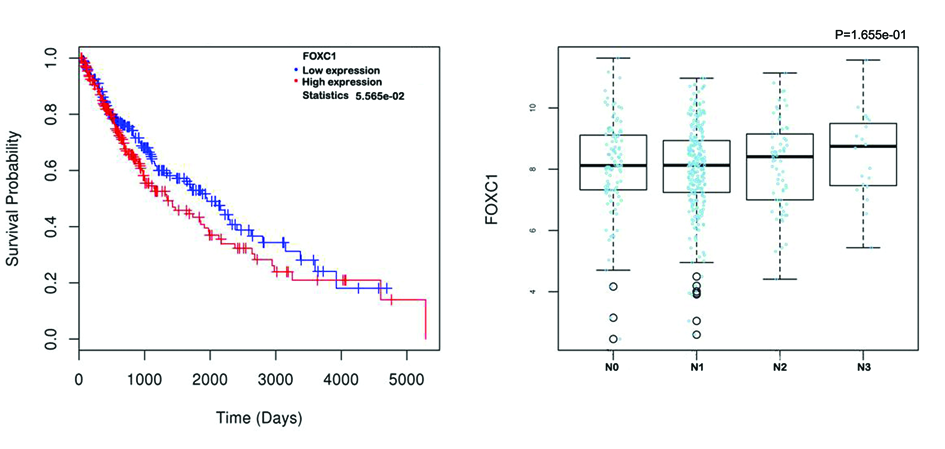

Supplement: Supplementary file 5 — Additional file 5: Figure S4. FOXC1 mRNA expression data were downloaded from the TCGA database containing 479 LUSC cancer tissues. Overall survival (OS) of patients in relation to FOXC1 expression status (high vs. low) was analyzed by the COX regression test. The relationship between FOXC1 expression and lymph node metastasis (N0-N3) was analyzed by Kruskal-Wallis test. [file 13046_2019_1478_MOESM5_ESM.tif]
